# A Case Report of Renal Infarcts Secondary to Segmental Arterial Mediolysis

**DOI:** 10.7759/cureus.58933

**Published:** 2024-04-24

**Authors:** Rebecca A Bowie, Adrianna D Clapp, Robyn L Reese, Christine Q Nguyen, Patricia Y Chipi

**Affiliations:** 1 Family Medicine, Mayo Clinic, Jacksonville, USA; 2 Division of Gerontology, Beth Israel Deaconess Medical Center, Harvard Medical School Multicampus Geriatric Fellowship Program, Boston, USA; 3 Hospital Internal Medicine, Mayo Clinic, Jacksonville, USA

**Keywords:** acute kidney injury, hypertension, segmental arterial mediolysis, renal artery dissection, renal infarct, flank pain

## Abstract

Flank pain is an exceptionally common presenting symptom in the emergency and primary care setting; however, most clinicians may not include a differential diagnosis of renal infarct (RI) due to the reported low incidence of this condition. Delayed diagnosis or treatment intervention for RI can have dire consequences for the patient including hypertension and longstanding renal impairment. In this report, we review a case of a previously healthy 39-year-old male presenting with flank pain, which after extensive workup, was revealed to be caused by renal infarction from a renal artery dissection secondary to segmental arterial mediolysis (SAM).

## Introduction

Flank pain is a common presenting symptom in the emergency department (ED) and can be caused by a variety of diseases [1]. The differential diagnosis for acute flank pain may be urinary (including nephrolithiasis, renal tumor, vesicoureteral reflux, pyelonephritis, perinephric abscess, trauma, and renal infarction (RI)) or extraurinary (including diverticulitis, appendicitis, and gallbladder disease) [2]. RI is a rare condition, with an estimated incidence in the ED of 0.004% to 0.007% [3]. RI develops when the renal artery blood flow is abruptly blocked, which leads over 95% of patients facing this condition to experience abdominal and/or flank pain [2,4]. Risk factors include atrial fibrillation, heart disease (infarction, valvular, or ischemic heart disease), endocarditis, vasculopathy, hematologic disease, trauma, and a hypercoagulable state [1,4]. Hypercoagulable states may be inherited (genetic causes such as Factor V Leiden and protein C deficiency) or acquired (surgery, immobilization, autoimmune condition, malignancy, inflammation, infection, and hormone-related including pregnancy, hormone replacement therapy, and contraception) [5]. Vascular pathology causes are often aortorenal such as atherosclerosis, aneurysm, dissection, fibromuscular dysplasia (FMD), and vasculitis, which can be in the setting of an underlying renal disease (such as nephrotic syndrome or glomerulonephritis) [6]. Although there are numerous underlying causes of RI, there are case reports where a cause is undetermined despite extensive investigations and is considered idiopathic [4].

RI is often misdiagnosed or diagnosed late because of the rarity of the condition and non-specific symptom presentation [4]. Studies report that approximately one-third of patients (27-32%) with RI develop long-term complications such as chronic kidney disease and a small proportion (2-5%) progress to end-stage renal disease [1]. Early identification is important in initiating treatment to minimize risks of long-term sequelae. We present a case of a 39-year-old male with acute flank pain who was found to have RI on a CT scan thought to be secondary to post-infectious hypercoagulability and later found to have a renal artery dissection secondary to segmental arterial mediolysis (SAM).

The preliminary findings of this case report were presented as a poster at the 2022 Florida Academy of Family Physicians (FAFP) Spring Forum on April 8, 2022. 

## Case presentation

A previously healthy 39-year-old male with a one-month history of ongoing abdominal pain and diarrhea presented to the ED with acute-onset right flank pain that started one hour prior. Early in the course of his illness, he was diagnosed with gastritis in the outpatient setting and was treated with a proton pump inhibitor with incomplete relief. Two weeks later, he presented with worsening symptoms to an outside hospital and was diagnosed with ascending diverticulitis visualized on CT abdomen/pelvis without contrast and was treated with a 10-day course of oral ciprofloxacin and metronidazole.

Approximately seven days into his antibiotic therapy and general improvement in his symptoms, he was able to advance his diet from clear liquid to low fiber. Despite this, he had sudden onset sharp right-sided flank pain and one episode of non-bloody, nonbilious emesis along with loose bowel movements after eating breakfast. Of note, he had minimal tobacco use in his teens but was a current non-smoker and had approximately one to four alcoholic drinks on weekdays and often more on weekends. He did not have a family history of cancer, hypercoagulability, or venous thromboembolism.

On arrival to the ED, his blood pressure was elevated at 164/84 mmHg; he had a heart rate of 84 beats per minute, respiratory rate of 18 breaths per minute, and temperature of 37.3°C. On physical examination, he was noted to have focal tenderness of the right mid-abdomen without rebound or guarding. Lab work showed mild thrombocytosis (372 x10(9)/L) on complete blood count (Table 1); creatinine was slightly elevated to 1.21 mg/dL (from 0.98 mg/dL one month prior) with mild hypokalemia (3.5 mmol/L) and hypochloremia (97 mmol/L) on comprehensive metabolic panel (Table 2). Lactate dehydrogenase (LDH) was elevated to 317 U/L. Urinalysis showed ketones, trace hemoglobin, mild leukocyte esterase, and elevated protein with normal-range red and white blood cells (Table 3).

**Table 1:** Patient’s complete blood count results on presentation to the emergency department. *Indicates value not in the normal range. MCV: mean corpuscular volume; MCH: mean corpuscular hemoglobin; MCHC: mean corpuscular hemoglobin concentration; RDW CV: red blood cell distribution width corpuscular volume; RDW SD: red blood cell distribution width standard deviation; L: liter; g/dL: gram per deciliter; fL: femtoliter; pg: picogram

Component	Lab value	Reference range and units
Leukocytes	5.9	3.4 - 9.6 x10(9)/L
Erythrocytes	4.35	4.35 - 5.65 x10(12)/L
Hemoglobin	13.6	13.2 - 16.6 g/dL
Hematocrit	39.2	38.3 - 48.6 %
MCV	90.1	78.2 - 97.9 fL
MCH	31.3	25.4 - 32.7 pg
MCHC	34.7	32.1 - 35.6 g/dL
RDW CV	11.6* (Low)	11.8 - 14.5 %
RDW SD	38.5	35.1 - 43.9 fL
Platelet count	372* (High)	135 - 317 x10(9)/L
Mean platelet volume	9.2	7.6 - 10.8 fL

**Table 2:** Patient’s comprehensive metabolic panel results on presentation to the emergency department. *Indicates value not in the normal range. mmol/L: millimoles per liter; mg/dL: milligrams per deciliter; mL/min/BSA: milliliters per minute per body surface area; U/L: units per liter; g/dL: gram per deciliter

Component	Lab value	Reference range and units
Sodium	137	135-145 mmol/L
Potassium	3.5* (low)	3.6-5.2 mmol/L
Chloride	97* (low)	98-107 mmol/L
Bicarbonate	28	22-29 mmol/L
Anion gap	12	7-15
Blood urea nitrogen (BUN)	7	8-24 mg/dL
Creatinine	1.21	0.74-1.35 mg/dL
Estimate glomerular filtration rate (eGFR)	75	mL/min/BSA
Calcium total	8.7	8.6-10 mg/dL
Glucose	97	70-140 mg/dL
Bilirubin, total	0.4	<1.2 mg/dL
Bilirubin, direct	0.1	0.0-0.3 mg/dL
Alanine aminotransferase (ALT)	25	7-55 U/L
Aspartate aminotransferase (AST)	29	8-48 U/L
Alkaline phosphatase	39* (low)	40-129 U/L
Total protein	6.6	6.3-7.9 g/dL
Albumin	4.3	3.5-5.0 g/dL
Lactate dehydrogenase (LDH)	317* (high)	122 - 222 U/L

**Table 3:** Patient’s urinalysis on presentation to the emergency department. *Indicates value not in the normal range. mg/dL: milligram per deciliter; hpf: high-power field

Component	Value	Reference range and units
Color	Yellow	Yellow
Clarity	Clear	Clear
Glucose	Negative	Negative, mg/dL
Ketones	Positive*	Negative
Hemoglobin (qualitative)	Trace*	Negative
Protein	30 mg/dL*	Negative, mg/dL
Nitrite	Negative	Negative
Bilirubin	Negative	Negative
Specific gravity	1.022	1.002-1.030
pH	6.5	5.0-8.0
Urobilinogen	Normal	Normal
Leukocyte esterase	Small*	Negative
White blood cells	1 hpf	Male 0-3 hpf; female 0-10 hpf
Red blood cells	1 hpf	0-2 hpf

**Figure 1:**
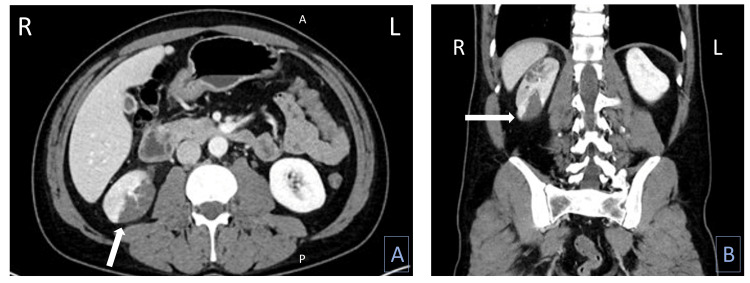
CT of abdomen/pelvis with IV contrast showing right upper and lower pole renal infarcts (arrow), (A) axial view and (B) coronal view. A: anterior; P: posterior; R: right; L: left

CT abdomen/pelvis with IV contrast demonstrated right upper and lower pole renal infarcts thought to be secondary to renal arterial thrombi/emboli without evidence of diverticulitis (Figure 1). Specifically, the report detailed a normal caliber abdominal aorta. The right renal artery was patent proximally for about 2.6 cm from its origin and then the artery became partially occluded and bifurcated; the right inferior pole renal artery was intermittently/partially opacified and then completely occluded distally; and the right upper pole renal artery was intermittently/partially opacified and then occluded distally. The left renal artery appeared patent and unremarkable and the bilateral renal veins were patent.

He was admitted to the family medicine hospital service for further workup and management. Interventional radiology and vascular surgery were consulted. No surgical or interventional procedures were recommended. He was promptly placed on anticoagulation via an unfractionated heparin infusion. A workup of the underlying cause included hypercoagulability (protein C activity, protein S activity, antithrombin activity, prothrombin G20210A mutation, and Factor V Leiden mutation), inflammatory (sedimentation rate (ESR) and C-reactive protein (CRP)), vasculitis (myeloperoxidase antibody, proteinase 3 antibody, and cryoglobulin), and endocrine lab studies (thyroid-stimulating hormone (TSH) and hemoglobin A1c), all of which resulted as normal or negative.

In addition, a cardiac workup with continuous cardiac monitoring, transthoracic echocardiogram (TTE), and transesophageal echocardiogram (TEE) were unremarkable. His course was complicated by acute kidney injury (creatinine increased to the nadir of 1.46 mg/dL) and hypertension, for which he required IV lactated ringers and was initiated on amlodipine with improvement. Ultimately, the patient's overall clinical picture improved with decreased abdominal pain, improved oral intake, and creatinine improved to 1.08 mg/dL. He was discharged on apixaban with a plan for continued additional outpatient workup and management. The leading diagnosis at this time was post-infectious hypercoagulability leading to RI.

Two weeks after discharge, the patient returned to the clinic with a few days' history of new epigastric pain, and a repeat CT abdomen/pelvis with IV contrast showed that the region of hypoperfusion in the lower pole of the right kidney was smaller; however, the region of hypoperfusion in the upper pole was larger compared with prior CT. There was concern for interval development of a 10 mm aneurysm or pseudoaneurysm proximal to an occluded segment of a right renal artery branch near the renal hilum.

**Figure 2:**
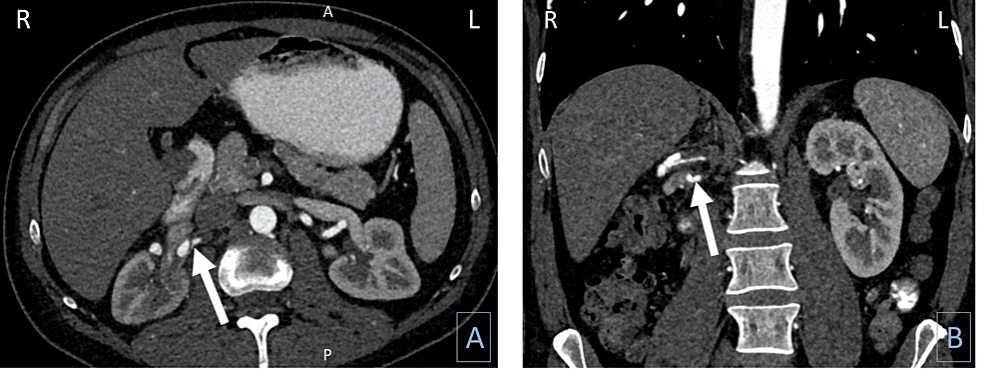
Repeat CT of abdomen/pelvis with IV contrast showing the dissection of the renal artery (arrow), (A) axial view and (B) coronal view. A: anterior; P: posterior; R: right; L: left.

An outpatient vascular surgery consult was conducted, where the images were reviewed, and it was determined that the patient's renal artery pathology appeared to be a complex flap dissection with reconstitution of normal arterial anatomy, which could have given the appearance of an aneurysm (Figure 2). On revisiting the previous CT scan from the ED, it appeared that the dissection was present at that time as well.

Rheumatology evaluated the patient, and he was tested for autoimmune causes (antinuclear (ANA) antibody, beta-2 glycoprotein 1 antibodies IgG and IgM, double-stranded DNA (dsDNA) antibody, SS-A/Ro antibody, SS-B/La antibody, Smith (Sm) antibody, ribonucleoprotein (RNP) antibody, anti-topoisomerase I (anti-Scl-70) antibodies, histidyl tRNA synthetase (Jo-1) antibodies, and complements C3 and C4), all of which resulted negative. The hepatitis panel and human immunodeficiency virus (HIV) were also tested and found to be negative. Nephrology continued to monitor the patient and losartan was added for better blood pressure control with a goal of <120/<80 mmHg.

The patient had a six-month return visit with vascular surgery with a repeat CT abdomen, which revealed SAM of the right renal artery with interval positive remodeling and mild residual undulation of the segmental branches. The radiologist noted this could mimic fibromuscular dysplasia (FMD). At 11 months from hospitalization, the patient was switched from apixaban to aspirin 325mg. During this time, he saw his primary care team a few times for recurrent abdominal discomfort and completed an esophagogastroduodenoscopy (showing reactive gastropathy) and colonoscopy (showing diverticulosis). One year after hospitalization, there was a primary care note indicating he no longer had symptoms. Given his full clinical improvement, a renal artery biopsy was not pursued, and the diagnosis of SAM was suspected based on clinical and imaging findings. 

## Discussion

This case exemplifies a common clinical complaint of flank pain, which was found to be the result of RI caused by renal artery dissection secondary to SAM. While our CT scan was aimed at evaluating for a renal etiology, RI was not high on the differential diagnosis list. The variability in the presentation of an RI is often the cause of failure to recognize this disease [7]. In fact, many renal infarcts are found incidentally on CT imaging while searching for other etiologies. Nonspecific symptoms like nausea, vomiting, and fever are often present with the chief complaint of unilateral flank pain and may prompt a clinician to explore more likely pathologies such as pyelonephritis or nephrolithiasis [7].

A 2021 retrospective observational study found the five most common clinical factors that could predict acute RI in the ED include male sex, age > 65 years old, smoking history, atrial fibrillation, and lack of hematuria [1]. Other studies have found proteinuria, elevated LDH, leukocytosis, CRP, and hematuria were predictors [1,3,4]. Hematuria versus lack of hematuria is a controversial risk factor in the literature; renal infarction can lead to glomerular and tubular damage resulting from tissue necrosis and result in hematuria. However, nephrolithiasis is a more common condition likely to present with hematuria, so its presence may not be a reliable or specific predictor for RI. Our patient was a young male who was a non-smoker with no history of atrial fibrillation. His urinalysis in the ED showed no hematuria; therefore, he only had two of the five most common clinical predictors. He did have an elevated LDH but no leukocytosis and was diagnosed by a CT scan.

Early diagnosis of RI and initiation of treatment have shown a positive effect on renal outcomes [8]. The primary goal is to restore perfusion when a thrombus is present, which may include vascular intervention, immediate anticoagulation, and strict blood pressure control [9]. In this case, the patient was diagnosed with RI upon arrival to the ED; he was started on a heparin infusion and transitioned to apixaban.

RI has many possible risk factors and therefore an extensive work-up including hypercoagulability, autoimmune, and cardiac studies is necessary in addition to assessing for connective tissue conditions such as Marfan syndrome or Ehlers-Danlos. Other considerations include family history, substance use, smoking history, and infectious causes. In this case, the working etiology of the RI was a thrombus in the setting of a post-infectious or inflammatory state from recently treated diverticulitis; however, upon further outpatient assessment and a multi-disciplinary approach with sub-specialists, it was discovered that the patient had evidence of renal artery dissection secondary to SAM.

SAM is a rare but serious vasculopathy of unknown etiology but is thought to be nonatherosclerotic and noninflammatory [10]. SAM often results in occlusion, stenosis, aneurysm, or dissection of abdominal aorta artery branches [10]. Based on the limited literature, there is no current consensus on diagnostic criteria and management. A 2019 literature review found that patients with SAM were more likely to be male, having a median age of 55, were tobacco users, and had hypertension and/or hyperlipidemia as a co-morbidity [10]. The most common vessel to be affected was the mesenteric artery followed by hepatic, celiac, renal, and splenic arteries, and the most common symptom was abdominal/flank pain [10]. The current treatment options for SAM depend on severity. The mortality rate for patients presenting emergently with arterial rupture and hemorrhage is estimated at 50% [11]. When there is hemodynamic instability, interventions may include coil embolization, abdominal organ surgery, or open arterial repair [10]. For non-urgent cases, a review of the literature found that patients were treated with blood pressure control, anticoagulation, and/or antiplatelet therapies [10]. Conservative management with close surveillance using magnetic resonance angiography (MRA) is recommended for these non-urgent cases to monitor disease progression [11].

The current gold standard for SAM diagnosis is artery biopsy; however, radiographic imaging combined with clinical findings is often used [11]. The literature reports that if feasible and safe, a tissue biopsy can help exclude other similar arteriopathies such as FMD [10]. FMD is more common in females aged 40-50 with a presentation that is less severe and sometimes asymptomatic compared to SAM, which is more common in males aged 50-60 with typically more severe and acute symptoms such as abdominal pain [10]. In review, our patient may have had a non-infectious cause of diverticulitis and perhaps it was SAM leading to abdominal pain and CT changes. The patient did not undergo a renal artery biopsy due to clinical improvement, but the diagnosis was suspected based on CT imaging and clinical presentation.

## Conclusions

RI, while rare, is an important diagnosis to include in the differential during the workup of acute flank and abdominal pain, which can have serious clinical implications with the potential for the development of long-standing renal impairment and hypertension. Prompt recognition can lead to timely intervention as well as avoidance of sequelae of more advanced renal hypoperfusion. RI has many potential risk factors, and therefore, a thorough workup for common etiologies is important. It is also important to consider that the etiology could be multi-factorial. Segmental arterial mediolysis is an important etiology to consider when evaluating a middle-aged male with RI as identifying this vasculopathy can guide acute and chronic management of the patient.
